# Supplementary material for: Content-rich biological network constructed by mining PubMed abstracts
Source: BMC Bioinformatics. 2004 Oct 8;5:147. doi: 10.1186/1471-2105-5-147 (PMC528731; doi:10.1186/1471-2105-5-147)
Supplement: Additional File 2 — The original results of the above study (non-essential files are deleted to keep the file size under the limit set by BMC bioinformatics). [file 1471-2105-5-147-S2.bz2 › chilibotAdditionalFile2/dip40/345ID9261146E4421/html/IL9_IL9R.html]

 


 **IL9** and **IL9R** 
  
Found 39 abstracts in PubMed, retrieved 39.  
 

 What does Google say? 
 PDF only 
| .edu only 

---

**Interactive relationship** (e.g. stimulation, inhibition, etc)

**Inhibitory relationship**- Human growth factor dependent cell line TF1, which lacks interleukin IL 9  [ **IL9** ]  receptors R and does not grow in IL 9  [ **IL9** ] , was transduced with a retroviral vector containing human IL 9R  [ **IL9R** ]  cDNA and a selection marker.  Ref: 10738974 J Hematother Stem Cell Res, 2000
- Sharing of IL 9R  [ **IL9R** ]  IL 2R components was furthermore suggested by inhibition of 125I IL 2 binding to primary AML cells with excess of unlabeled IL 9  [ **IL9** ] .  Ref: 8630406 Blood, 1996
**Stimulatory relationship**- Moreover, colony formation by IL 9R  [ **IL9R** ]  transduced cells was more sensitive to stimulation with lower doses of IL 9  [ **IL9** ]  and Epo.  Ref: 8971379 Bone Marrow Transplant, 1996
- An increased number and size of burst forming units erythroid BFU E like colonies were detected from IL 9R  [ **IL9R** ]  transduced TF1 cells, compared with mock transduced cells, in response to erythropoietin EPO and IL 9  [ **IL9** ] .  Ref: 10738974 J Hematother Stem Cell Res, 2000
**Neutral relationship**- Cosmids containing the human IL 9  [ **IL9** ]  receptor R gene  **IL9R**  have been isolated from a genomic library using the  **IL9R**  cDNA as a probe.  Ref: 8666384 Genomics, 1995
- The potential roles of multiple interactions between IL 9  [ **IL9** ] , IL 9R  [ **IL9R** ]  and HTLV I in the monoclonal expansion and transformation of MT 2 cells are explored.  Ref: 8622860 Oncogene, 1996
- Interleukin 9  [ **IL9** ]  IL 9  [ **IL9** ]  exerts its pleiotropic effects through the IL 9  [ **IL9** ]  receptor IL 9R  [ **IL9R** ]  complex, which consists of the IL 9R  [ **IL9R** ]  alpha chain, which determines the cytokine specificity, and the IL 2 receptor gamma chain.  Ref: 10642536 Biochem J, 2000
- Interleukin 9  [ **IL9** ]  IL 9  [ **IL9** ]  exerts its pleiotropic effects through the IL 9  [ **IL9** ]  receptor IL 9R  [ **IL9R** ]  complex that consists of the ligand specific IL 9R  [ **IL9R** ]  alpha chain, and the IL 2R gamma chain.  Ref: 10486269 Biochem Biophys Res Commun, 1999

**Non-interactive relationship** (e.g. studied together, co-existance, homology, etc.)

- Colony formation by BFU E transduced with the IL 9R  [ **IL9R** ]  gene and grown without selection in G418 and in the presence of erythropoietin Epo and interleukin IL 9  [ **IL9** ]  was significantly increased up to three fold and the size of the erythroid colonies was significantly increased 50 100% compared to colony formation by mock virus transduced cells.  Ref: 8971379 Bone Marrow Transplant, 1996
- OBJECTIVE To investigate the contribution of IL 9  [ **IL9** ]  to the pathogenesis of asthma, we examined the expression of IL 9  [ **IL9** ]  and its receptor IL 9R  [ **IL9R** ]  in bronchial tissue from subjects with atopic asthma n = 10, chronic bronchitis n = 11, and sarcoidosis n = 9 and from atopic n = 7 and nonatopic n = 10 healthy control subjects.  Ref: 10629460 J Allergy Clin Immunol, 2000
- Since our genetic analysis of the 5q cytokine cluster did not support a genetic role for the  **IL9**  gene, we became interested in the  **IL9**  receptor gene  **IL9R**  in the pseudoautosomal region.  Ref: 11039580 Eur J Hum Genet, 2000
- The IL 9  [ **IL9** ]  receptor gene  **IL9R**  genomic structure, chromosomal localization in the pseudoautosomal region of the long arm of the sex chromosomes, and identification of  **IL9R**  pseudogenes at 9qter, 10pter, 16pter, and 18pter.  Ref: 8666384 Genomics, 1995
- Analysis of IL 9R  [ **IL9R** ]  alpha subunit expression on 18 T cell clones revealed that IL 9  [ **IL9** ]  responsiveness was directly proportional to the expression of the high affinity receptor.  Ref: 10888117 J Interferon Cytokine Res, 2000
- IL 9  [ **IL9** ]  and IL 9R  [ **IL9R** ]  positive cells were identified with immunocytochemistry, and HCLCA1 expression was detected by means of in situ hybridization with cRNA probes.  Ref: 11842292 J Allergy Clin Immunol, 2002
- A search for gene s associated with anti human immunodeficiency virus type 1 HIV 1 activity of CD8 T cells was attempted using molecular cloning and the relation between the anti HIV activity of CD8 T cells and the interleukin 9  [ **IL9** ]  receptor alpha chain IL 9R  [ **IL9R** ]  alpha mRNA expression from the cDNA clones obtained was examined.  Ref: 9645243 Acta Virol, 1998
- To examine the interaction between IL 9  [ **IL9** ]  and eosinophils, we evaluated mature peripheral blood eosinophils for their expression of the specific alpha subunit of the IL 9  [ **IL9** ]  receptor IL 9R  [ **IL9R** ]  alpha.  Ref: 10979962 Blood, 2000
- Human T cell lines and clones, derived from PBMC by stimulation with PHA, IL 2, and irradiated allogeneic PBMC, were found to express a strong IL 9R  [ **IL9R** ]  message at the RNA level and to proliferate in the presence of IL 9  [ **IL9** ] , irrespectively of their CD4 or CD8 phenotype.  Ref: 8454846 J Immunol, 1993
- IL 9  [ **IL9** ] , IL 9R  [ **IL9R** ] , and hCLCA1 expression was determined by immunocytochemical study.RESULTSWe demonstrated significant increases in IL 9  [ **IL9** ] , IL 9R  [ **IL9R** ] , and hCLCA1 immunoreactivity in the mucosa of patients with cystic fibrosis compared with that found in control subjects P < ;.  Ref: 12782818 Laryngoscope, 2003
- Prior to superinfection, the cells express neither IL 9  [ **IL9** ]  nor IL 9R  [ **IL9R** ] .  Ref: 7966560 J Virol, 1994
- OBJECTIVE We sought to examine the expression of HCLCA1 in bronchial biopsy specimens of asthmatic subjects compared with those of control subjects and to demonstrate its relationship with IL 9  [ **IL9** ] , IL 9  [ **IL9** ]  receptor IL 9R  [ **IL9R** ] , and markers of mucus production.  Ref: 11842292 J Allergy Clin Immunol, 2002
- In this study, we evaluated expression of the IL 9R  [ **IL9R** ]  and the effect of IL 9  [ **IL9** ]  on human PMNs.  Ref: 11160343 J Immunol, 2001
- Reverse transcriptase polymerase chain reaction of BEAS 2B, HBE4 E6 E7, and primary cells from two subjects revealed messenger RNA for IL 9  [ **IL9** ]  receptor IL 9R  [ **IL9R** ]  alpha but not in BEAS 2B cells.  Ref: 11588013 Am J Respir Cell Mol Biol, 2001
- The IL 9  [ **IL9** ]  receptor IL 9R  [ **IL9R** ]  belongs to the haemopoietic receptor superfamily and its signal transduction involves mainly the Janus kinase signal transducer and activator of transcription JAK STAT pathway.  Ref: 11115404 Biochem J, 2001
- We constructed a retroviral vector, pLhIL 9RSN, containing CDNA encoding the human interleukin 9  [ **IL9** ]  receptor IL 9R  [ **IL9R** ]  along with a neomycin phosphotransferase gene Neo.  Ref: 8971379 Bone Marrow Transplant, 1996
- TF1 cells stably expressing IL 9R  [ **IL9R** ]  and responding to IL 9  [ **IL9** ]  can serve as a cell line model to study the intracellular signals mediating IL 9  [ **IL9** ]  induced erythroid cell proliferation and differentiation.  Ref: 10738974 J Hematother Stem Cell Res, 2000
- In this report, we present evidence that the vast majority > 90% of the IL 2 independent lines established from polytropic or xenotropic virus infected 4437A cells carry provirus insertions in the 3 untranslated region of the IL 9  [ **IL9** ]  receptor gene Gfi 2 for growth factor independence 2 IL 9R  [ **IL9R** ] .  Ref: 7966560 J Virol, 1994
- The establishment of an IL 9  [ **IL9** ]  dependent autocrine loop was sufficient to render the cells IL 2 independent, as suggested by the finding that 4437A cells, expressing a stably transfected Gfi 2 IL 9R  [ **IL9R** ]  construct, do not require IL 2 when maintained in IL 9  [ **IL9** ]  containing media.  Ref: 7966560 J Virol, 1994
- CONCLUSIONSIncreased expression of IL 9  [ **IL9** ]  and IL 9R  [ **IL9R** ] , as well as upregulation of hCLCA1, in mucus overproducing epithelium of patients with cystic fibrosis supports the hypothesis that IL 9  [ **IL9** ]  contributes to mucus overproduction in cystic fibrosis.  Ref: 12782818 Laryngoscope, 2003
- IL 9  [ **IL9** ]  responsiveness was also dependent on long term culturing because neither freshly isolated nor 3 day phytohemagglutinin PHA stimulated peripheral blood lymphocytes PBL expressed IL 9R  [ **IL9R** ]  alpha.  Ref: 10888117 J Interferon Cytokine Res, 2000
- METHODS Bronchial biopsy specimens were examined for the presence of IL 9  [ **IL9** ]  and IL 9R  [ **IL9R** ]  protein and messenger RNA mRNA by immunocytochemistry and in situ hybridization, respectively.  Ref: 10629460 J Allergy Clin Immunol, 2000
- Interleukin 9  [ **IL9** ]  receptor IL 9R  [ **IL9R** ]  complex consists of a ligand specific alpha chain and IL 2R gamma chain.  Ref: 9261146 J Biol Chem, 1997
- IL 9  [ **IL9** ]  is not a neutrophil agonist as demonstrated by the inability to modulate the tested functions responses that correlate with lack of the IL 9R  [ **IL9R** ]  component, namely, IL 9Ralpha.  Ref: 9743609 Clin Immunol Immunopathol, 1998
- By analysing a genomic clone, we identified the integration site of a single HTLV I long terminal repeat LTR in the interleukin 9  [ **IL9** ]  IL 9  [ **IL9** ]  receptor IL 9R  [ **IL9R** ]  gene.  Ref: 8622860 Oncogene, 1996
- We sought to examine the expression of IL 9  [ **IL9** ] , interleukin 9  [ **IL9** ]  receptor IL 9R  [ **IL9R** ] , and hCLCA1 in the upper airway of patients with cystic fibrosis in comparison to healthy control subjects and to demonstrate the relationship of IL 9  [ **IL9** ] , IL 9R  [ **IL9R** ] , and hCLCA1 expression with mucus production.STUDY DESIGNProspective design.METHODSBiopsy samples from nasal polyps of four patients with cystic fibrosis, nasal mucosa of six patients with cystic fibrosis, sinus mucosa of eight patients with cystic fibrosis, and nasal mucosa of six healthy control subjects were stained with periodic acid Schiff PAS to identify mucus glycoconjugates.  Ref: 12782818 Laryngoscope, 2003
- We show that the interleukin 9  [ **IL9** ]  IL 9  [ **IL9** ]  receptor gene  **IL9R** , which is located within the human Xq Yq homology region, maps to the murine chromosome 11.  Ref: 9002663 Hum Mol Genet, 1997
